# Computational infrared and Raman spectra by hybrid QM/MM techniques: a study on molecular and catalytic material systems

**DOI:** 10.1098/rsta.2022.0234

**Published:** 2023-07-10

**Authors:** Jingcheng Guan, You Lu, Kakali Sen, Jamal Abdul Nasir, Alec W. Desmoutier, Qing Hou, Xingfan Zhang, Andrew J. Logsdail, Gargi Dutta, Andrew M. Beale, Richard W. Strange, Chin Yong, Paul Sherwood, Hans M. Senn, C. Richard A. Catlow, Thomas W. Keal, Alexey A. Sokol

**Affiliations:** ^1^ Department of Chemistry, University College London, London WC1H 0AJ, UK; ^2^ STFC Scientific Computing, Daresbury Laboratory, Keckwick Lane, Daresbury, Warrington WA4 4AD, UK; ^3^ Cardiff Catalysis Institute, School of Chemistry, Cardiff University, Park Place, Cardiff CF10 3AT, UK; ^4^ Research Complex at Harwell, Rutherford Appleton Laboratory, Harwell Oxford, Didcot OX11 0FA, UK; ^5^ Department of Physics, Balurghat College, Balurghat 733101, West Bengal, India; ^6^ School of Life Sciences, University of Essex, Wivenhoe Park, Colchester, Essex CO4 3SQ, UK; ^7^ Department of Chemistry, Lancaster University, Lancaster LA1 4YB, UK; ^8^ School of Chemistry, University of Glasgow, Joseph Black Building, Glasgow G12 8QQ, UK; ^9^ Institute of Photonic Chips, University of Shanghai for Science of Technology, Shanghai 201512, People’s Republic of China

**Keywords:** vibrational spectroscopy, infrared, Raman, embedded cluster, QM/MM, ChemShell

## Abstract

Vibrational spectroscopy is one of the most well-established and important techniques for characterizing chemical systems. To aid the interpretation of experimental infrared and Raman spectra, we report on recent theoretical developments in the ChemShell computational chemistry environment for modelling vibrational signatures. The hybrid quantum mechanical and molecular mechanical approach is employed, using density functional theory for the electronic structure calculations and classical forcefields for the environment. Computational vibrational intensities at chemical active sites are reported using electrostatic and fully polarizable embedding environments to achieve more realistic vibrational signatures for materials and molecular systems, including solvated molecules, proteins, zeolites and metal oxide surfaces, providing useful insight into the effect of the chemical environment on the signatures obtained from experiment. This work has been enabled by the efficient task-farming parallelism implemented in ChemShell for high-performance computing platforms.

This article is part of a discussion meeting issue ‘Supercomputing simulations of advanced materials’.

## 1. Introduction

Vibrational spectroscopy techniques are commonly employed in studies of materials used by the electronic, pharmaceutical, cosmetic, food and many other industries [1]. Infrared (IR) and Raman techniques are useful for determination of molecular building blocks of materials, characterization and identification of molecular species and their bonding [2]. The vibrational fingerprints assist experimental researchers to analyse reaction intermediates and discover new chemical mechanisms [3–7]. IR spectroscopy is based on the absorption of light by matter in the IR spectral region. Raman spectroscopy provides complementary information on molecular vibrations using inelastic photon scattering [8]. Interpretation of spectroscopic signatures is, however, not straightforward under in situ chemical conditions. Thus, a connection between the experimental findings and theoretical models needs to be established to understand various chemical processes by exploiting the power of modern computation.

Theoretical descriptions of IR and Raman processes have been under continuous development since the last century [9–18]. Based on the conventional harmonic approximation of a potential energy surface (PES), computation of vibrational spectra has now become a standard task in quantum chemistry codes as density functional theory (DFT) methods have matured and proved to be successful in efficiently predicting the electronic structure of most molecular and condensed matter systems. Although analytical second and higher derivatives are, in principle, available in many computational chemistry codes for some forcefield and electronic structure methods, more generally, force constants and frequencies of vibrational motion are readily obtained from nuclear gradients at stationary points on the PES. At each point, the time-independent Schrödinger equation under the Born–Oppenheimer approximation is solved for energy and gradients, alongside other electronic properties, including the dipole moment and polarizability. More advanced methods, beyond the harmonic approximation, have also been developed and employed to account for anharmonicity and nuclear quantum effects observed in vibrational spectra [15–17,19–24].

Hybrid quantum mechanical and molecular mechanical (QM/MM) approaches provide an accurate description of local chemistry, with particular strength in modelling large complex systems, while maintaining the flexibility of choice for the level of quantum mechanical theory applied to different subregions of the model. In QM/MM calculations, a region of chemical interest is modelled using high-level quantum mechanical theories to obtain an accurate description of the electronic structure, while the surrounding system is described by classical molecular mechanical forcefields, which are much less computationally expensive. The interface region between the QM and MM boundaries, which will be discussed in §2, is defined according to the nature of bonding of a chemical system being modelled.

For most extended systems, due to high computational cost and/or lack of necessary facilities in computer codes of choice, the impact of the chemical environment on vibrational signatures observed in IR and Raman spectra is not widely studied. QM/MM approaches are especially efficient in modelling local chemical properties without sacrificing overall accuracy at active sites, while avoiding treating the whole large system quantum mechanically. Previously, progress has been achieved on local vibrational spectra [25,26]. However, polarizable environmental effects within various types of materials have not been sufficiently addressed. Therefore, the description of local vibrations and the IR and Raman processes subject to environmental effects are the focus of this study on complex molecular systems and heterogeneous catalysts.

Many software packages are capable of calculating vibrational frequencies [27–32]. IR and Raman signatures are also available at both molecular QM and MM levels of theory [27–29,32]. There are also some more recent developments for periodic systems [33,34]. For hybrid QM/MM coupling schemes based on mechanical and electrostatic embedding, such spectra can also be calculated [35–43]. However, there are fewer studies exploring the calculation of IR and Raman intensities using fully polarizable hybrid QM/MM methods [44–48].

In this work, we report on the development and implementation of functionality for calculation of IR and Raman spectra in ChemShell, a scriptable computational chemistry environment with an emphasis on the multiscale modelling of complex chemical systems using QM/MM techniques [49–51]. This work has been carried out in the recently redeveloped Python-based version of ChemShell (‘Py-ChemShell’) [49], which offers a modern open-source platform for the development of advanced embedding methods. Functionality for calculating harmonic IR and Raman spectroscopic signatures has been implemented in Py-ChemShell for both QM-only and hybrid QM/MM approaches at all levels of embedding, targeting multiscale modelling with chemical accuracy and generating useful chemical insights for collaborative computational and experimental research work.

The outline of this article is as follows: in §2, a brief overview of hybrid QM/MM approaches is given with a focus on electrostatic and polarizable embedding and the calculation of vibrational properties. Details of the QM/MM test case models are also provided. In §3, results are provided for the example systems including an amino acid molecule in a water droplet explicit solvent model, a haem protein in solution, ammonia probing zeolite acidity and catalytic ZnO surfaces.

## 2. Methodology of QM/MM modelling

This section starts with a brief discussion of the additive QM/MM model used in this work [50] and QM/MM calculations of classical vibrational frequencies and normal modes using the harmonic approximation to the PES around equilibrium. The vibrational problem is further recast in the form of wave mechanics, by which theoretical IR and Raman intensities are derived [9]. Details of the QM/MM models used for each of the four applications are provided at the end of the section.

### (a) Harmonic vibrational calculations using QM/MM methods

The total energy of a QM/MM system is composed of three major parts: the QM energy of the inner region, the MM energy of the environment and the interaction between the respective two parts of the model, i.e.

2.1 $$E_{\text{total}}=E_{\text{QM}}+E_{\text{MM}}+E_{\text{QM-MM}},$$

where the QM energy is calculated in this study using DFT, and the MM energy is calculated with appropriate forcefields. In practice, factors such as prediction accuracy, efficiency and accessibility of the model are the main concerns when choosing specific levels of theory for the QM and MM parts.

All of our calculations are performed using electrostatic or higher levels of embedding. In the electrostatic embedding scheme, the Coulombic potential from the MM part upon the QM region is represented in the QM Hamiltonian in the form of point charges, resulting in polarization of the QM region by the MM environment, while external forces on the MM region coming from the QM region are obtained using gradients on the point charges that represent MM centres. In the polarizable embedding approach, used for the ZnO test case as described later, a shell model [52] is additionally employed to mimic polarization of atomic valence electrons. Shell relaxation is carried out modelling response of MM centres to changes in the electronic structure of the active QM region. Further, the charge density of the QM region should adapt to the relaxed shells leading to an iterative procedure that continues until a balance of charges in the two regions is reached.

For vibrational calculations in QM/MM approaches, the PES is approximated using a limited number of nuclear degrees of freedom; with selection of a vibrational chemically active region, the PES is expanded in terms of Cartesian coordinates of N vibrating active atoms, resulting in 3N vibrational modes. Due to a potentially strong interaction of the active region with its environment, nominally translational and rotational motions change their nature and should be treated as normal vibrations. The main embedding effects on the vibrational active region come from the polarizable surrounding via electrostatic interactions. The vibrational coupling of environmental phonons and motions at a local site is considered to have minimum impact on vibrations of chemically active sites modelled in this work. The calculation of classical vibrational modes at a harmonic level is implemented in Py-ChemShell via the integrated DL-FIND geometry optimization library [53] using a finite-difference approximation of the dynamical matrix.

### (b) IR intensities calculations using QM/MM approaches

IR absorption intensity is determined by the transition probability per unit time, $B_{m\to n}\rho (\nu_{mn})$, for states m and n, where following time-dependent perturbation theory [54]

2.2 $$B_{m\to n}=\frac{8\pi^{3}}{3h^{2}}\{\mu_{x_{mn}}^{2}+\mu_{y_{mn}}^{2}+\mu_{z_{mn}}^{2}\}$$

is the Einstein’s coefficient of absorption, *h* is Planck's constant, $\rho (\nu_{mn})$ is the radiation density at light frequency $\nu_{mn}$ and $\mu_{x_{mn}}=\langle m|{\hat{\mu }}_{x}|n\rangle$ is the transition dipole moment in the Cartesian direction x, with transition dipole moments along y and z directions defined similarly. The dipole moment operator is defined as a multiplication by a factor operator, where the factor is the dipole moment calculated as a function of nuclear coordinates and electronic charge density. The IR intensity calculation implemented in Py-ChemShell omits the experimental set-up form factor and is based on the intrinsic dipole moments of the molecular structure itself. In the harmonic approximation, the initial and final vibrational states are defined over normal mode coordinates. Using a Taylor expansion for the dipole moment along a normal mode and terminating on the linear term [9], sometimes called the double harmonic approximation [10]:

2.3 $$\mu_{x_{mn}}\approx \frac{\partial \mu_{x}}{\partial Q_{i}}\langle m|Q_{i}|n\rangle ,$$

where $\partial \mu_{x}/\partial Q_{i}$ is the derivative of the dipole moment along mode $Q_{i}$ calculated at the equilibrium position. In contrast to typical molecular QM codes, these derivatives are calculated in Py-ChemShell numerically, which enables complex polarizable embedding models to be used in the study of advanced materials.

In a hybrid QM/MM model, a finite QM region is embedded within an extended MM environment. Following the Born–Oppenheimer approximation, fast electronic polarization of the extended environment is modelled as mentioned earlier by balancing mutual polarization of the QM and MM regions. The calculated dipole moment, therefore, accounts for a change in the environment in response to vibrations in the chemically active region, and the dipole moment assumes the form:

2.4 $$\mu =-e\int r\rho (r)d^{3}r+e\sum_{\alpha }Z_{\alpha }R_{\alpha }+\sum_{i}z_{i}R_{i},$$

where ρ is the self-consistent charge density calculated in the presence of environmental point charges, r represents the electronic coordinate, e is the electron charge, $Z_{\alpha }$ are the charges of nuclei in the QM region, $R_{\alpha }$ are the positions of the nuclei, $z_{i}$ are the effective point charges of the MM environment and $R_{i}$ are the point charge positions. Thus, the calculated IR intensity incorporates not only interaction with environment but also its dielectric response.

### (c) Raman intensities calculations using QM/MM approaches

In a similar way to the IR intensity, the Raman intensity is defined as Raman scattering probability per unit time and is proportional to the Raman scattering factor (or Raman activity), $S^{\text{Raman}}$, determined by average transition polarizability of active centres between states m and n [10]:

2.5 $$S^{\text{Raman}}=45\alpha^{2}+7\beta^{2},$$

where

2.6 $$\alpha =\frac{1}{3}(\langle m|{\hat{\alpha }}_{xx}|n\rangle +\langle m|{\hat{\alpha }}_{yy}|n\rangle +\langle m|{\hat{\alpha }}_{zz}|n\rangle )$$

is the average isotropic transition polarizability, and

2.7 $$\begin{array}{rl} \beta^{2} & \;=\frac{1}{2}[{\left(\langle m|{\hat{\alpha }}_{xx}|n\rangle -\langle m|{\hat{\alpha }}_{yy}|n\rangle \right)}^{2}+{\left(\langle m|{\hat{\alpha }}_{yy}|n\rangle -\langle m|{\hat{\alpha }}_{zz}|n\rangle \right)}^{2}+{\left(\langle m|{\hat{\alpha }}_{zz}|n\rangle -\langle m|{\hat{\alpha }}_{xx}|n\rangle \right)}^{2}\\ & \;\;+6({\left\langle m|{\hat{\alpha }}_{xy}|n\right\rangle }^{2}+{\left\langle m|{\hat{\alpha }}_{xz}|n\right\rangle }^{2}+{\left\langle m|{\hat{\alpha }}_{yz}|n\right\rangle }^{2})] \end{array}$$

is the average anisotropic transition polarizability. The coefficient of proportionality between the Raman intensity and scattering factor is determined by the experimental set-up and the density of scattering centres. Following the double harmonic approximation, components of the transition polarizability along mode $Q_{i}$ are calculated as follows [9]:

2.8 $$\left\langle m|{\hat{\alpha }}_{xy}|n\rangle \approx \frac{\partial \alpha_{xy}}{\partial Q_{i}}\langle m|Q_{i}|n\right\rangle$$

and similarly for the other transition terms mentioned earlier. Since sometimes the transition moments are omitted from the calculation of the polarizabilities [28], Py-ChemShell provides both scaled and unscaled values of the scattering factors. The derivatives of the polarizability in our implementation are calculated numerically, similar to the derivatives of the dipole moment earlier, including the MM electronic response in the adiabatic approximation.

The transition polarizability tensor entering equations (2.5)–(2.8) depends on the frequency of the radiation. In this work, the NWChem software package [28] is employed as a QM driver. In NWChem, time-dependent density functional response theory is used for the calculation of frequency-dependent molecular polarizabilities [55,56]. Following the embedding procedure, the response of the electronic structure perturbed by the incident light is thus calculated in the presence of fixed point charges of the MM environment. For polarizable embedding based on the adiabatic approximation, mutual polarization between the QM and MM parts, however, does not account for response to the radiation-induced perturbation—the radiation is only applied to the QM region in the presence of fixed point charges. To improve on this approximation, more advanced embedding techniques are needed that include frequency-dependent response of the MM system in the calculation of electronic excited states localized in the embedded QM region.

For resonance Raman (RR) calculations, the resonant light wavelength that corresponds to an electronic transition(s) of interest must be first determined using the time-dependent density functional theory (TDDFT) approach implemented in NWChem (or alternatively any other available QM driver) [57,58]. The polarizability tensor close to the resonance is obtained using the method of Jensen *et al.* [12]. The finite lifetime of the excited state is accounted for using an empirical damping parameter as implemented in NWChem (0.006 a.u. in our work) [12,56,59]. As proposed by Jensen *et al.*, the calculation of intensity for resonance and non-resonance (normal) Raman scattering in ChemShell thereafter follows the same method as described earlier [12].

### (d) Implementation of IR and Raman signatures in ChemShell

This section focuses on implementation of the methodology discussed earlier for the calculation of IR and Raman intensities in the Py-ChemShell environment. The work builds on existing functionality in DL-FIND, which can calculate vibrational frequencies in the harmonic approximation, with elements of the Hessian matrix evaluated numerically as the first differences of analytical gradients in mass-weighted coordinates, using two-point central differences. We also make use of the task-farming parallelization framework in Py-ChemShell which has been used to accelerate numerical Hessian matrix calculations [60]. The previously developed DL-FIND design has been exploited here to support numerical evaluation of the first derivatives of the dipole moment and polarizability tensor along vibrational normal modes that appear in equations (2.3) and (2.8). For this purpose, the derivatives of the dipole and polarizability calculated first in mass-weighted Cartesian coordinates are projected on the normal modes using the chain rule.

**Figure 1.**
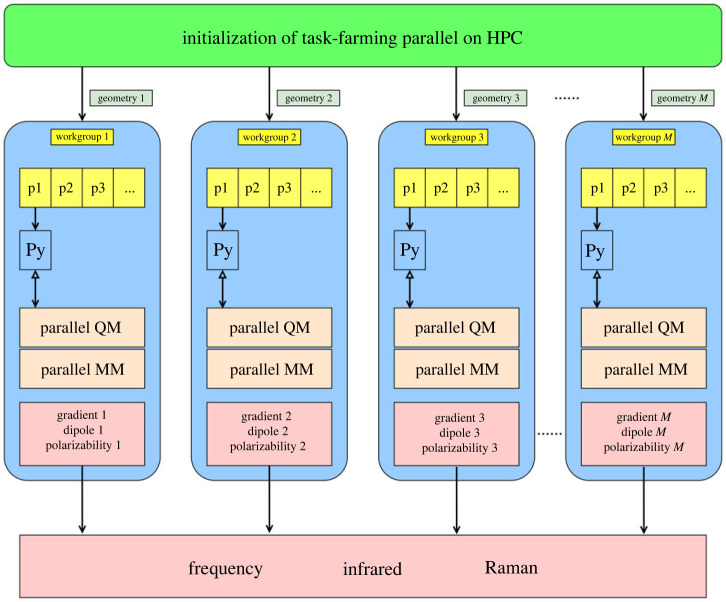
Schematic of the task-farming parallelization framework for the calculation of IR and Raman spectra in Py-ChemShell. The Hessian matrix, derivatives of dipoles and polarizabilities are calculated using two-point finite central differences. The single-point (SP) jobs for each Cartesian coordinate are distributed to task-farming work groups by the DL-FIND module. Each work group receives one SP geometry at a time and calls back the Python environment to execute interfaced QM and MM programs in parallel. Upon completion, the QM/MM results, including energy, gradient, dipole moment, and polarizability tensor of a structure, are returned to the task engine for calculating frequencies, normal modes, IR and Raman intensities. In the spectra calculations, 3N SP geometries are distributed to M work groups, where N represents the number of vibrational active atoms in a QM/MM structure. (Online version in colour.)

The task-farming infrastructure in ChemShell accelerates the calculation of IR and Raman spectra by distributing the finite-difference single-point calculations to a set of work groups, which can be run simultaneously. The time to solution for calculations of IR and Raman spectra, therefore, can be efficiently parallelized up to a maximum of the number of nuclear degrees of freedom. Figure 1 illustrates how the task-farming parallelization scheme is applied to IR and Raman calculations in Py-ChemShell.

Implementing the IR and Raman calculations with the DL-FIND module results in a flexible, modular framework for vibrational spectroscopy, which can be used with any of the QM packages interfaced to ChemShell, including NWChem [28], GAMESS-UK [27], FHI-aims [61], LSDalton [32] and others, controlled by a common Python interface. The computational IR and Raman procedures are then started in the Py-ChemShell environment by simply specifying the key words ir=True and raman=True given to an instance of the object Task.

Vibrational signatures of a group of 32 gas-phase molecules were calculated with the newly implemented IR and Raman routines in Py-ChemShell and used as a benchmark, with results provided in the electronic supplementary material. The test has been carried out regarding three different vibrational quantities, i.e. frequency, IR intensity and Raman intensity. Results obtained with the ChemShell routines are in good agreement with results calculated using the NWChem quantum chemistry code [28,56].

### (e) Hybrid QM/MM model for solvated histidine

The histidine molecule studied was the δ-protonated zwitterion, initially described by the CHARMM36m forcefield [62] and placed in a TIP3P [63] water cube of side length about 44 Å (padding by 20 Å in each direction around the histidine molecule). The system was equilibrated using NAMD [64] interfaced to Py-ChemShell. The MD simulations employed Langevin dynamics with periodic boundary conditions at 300 K. Long-range electrostatics were treated by the Particle Mesh Ewald method. The cut-off for non-bonding interactions was set at 12 Å with a smooth switching function turned on from 10 Å. Firstly, 10 000 steps of energy minimization were performed to relax any unphysical contacts, then a 200 ps classical NVT molecular dynamics (MD) simulation was performed with the histidine molecule fixed. Then another 10 000 steps of energy minimization were carried out, followed by a 500 ps NPT MD with all atoms relaxed. Finally, the production NPT MD was run for 1 ns without restraints to ensure full equilibrium. From this trajectory, snapshots were taken out at intervals of 50 ps and one of them randomly chosen for calculating the vibrational spectra.

The QM/MM water droplet model was built by cutting a sphere with a radius of 15 Å centred on the histidine molecule. The resulting system contained 1559 atoms (20 histidine atoms and 513 water molecules). We included the histidine and the 56 closest surrounding water molecules in the QM region, resulting in a total of 188 QM atoms. QM/MM calculations were performed by Py-ChemShell using NWChem [28] and DL\_POLY [65] for the DFT and molecular mechanics calculations, respectively. The density functional B3LYP [66,67] with a def2-SVP basis set [68,69] was used for the QM calculations. The water molecules in the MM region were described with the TIP3P [63] water model. The system was optimized at the QM/MM level using the DL-FIND [53] module before calculating the vibrational spectra. For the spectroscopic calculations, the vibrationally active region was restricted to the histidine molecule (20 atoms), and hence, 60 normal modes were computed. No scaling was applied to the calculated vibrational frequencies to directly assess performance against experimental results.

### (f) Hybrid QM/MM model for *Ax*CYTcp

The coordinates of the six-coordinate CO-bound (6cCO) form of the haem protein cytochrome c${\;}^{\prime }$ from the denitrifying bacterium *Alcaligenes xylosoxidans* (*Ax*CYTcp) were taken from the crystal structure with PDB ID 3ZWI [70]. A solvated model of the 6cCO system was generated using a new biomolecular solvation workflow in Py-ChemShell, full details of which will be published separately. Protonation states were assigned at pH 7 and hydrogen atoms added using the PDB2PQR [71] and PROPKA [72] programs. The system was solvated by padding 15 Å of equilibrated TIP3P [63] water in all the three directions and neutralizing the whole system by adding one ${\mathrm{Cl}}^{-}$ ion at a random vacant position. Explicit all-atom MD simulations to equilibrate the full system were run with NAMD [64] using the CHARMM36 forcefield. The MD simulations used the same parameters as for histidine.

The system was initially subjected to 5000 steps of conjugate gradient (CG) minimization to eliminate any unphysical contacts. Then, the water and ion were equilibrated in an NVT ensemble, keeping the protein fixed for 2 ns. This was followed by 5000 steps of CG minimization and 5 ns equilibration under an NPT ensemble keeping the backbone harmonically restrained (force: $5\;{\mathrm{kcal}/\mathrm{mol}/\text{Å}}^{2}$). The simulation was continued for another 30 ns with removal of backbone restraints but retaining of constraints for the haem, CO ligand and the proximal His120. In the NPT simulations, the pressure was maintained with the Langevin piston method.

A randomly chosen snapshot from the last 30 ns of NPT run was taken for subsequent QM/MM calculations. A QM/MM model consisting of the full protein and any water molecules within 7 Å of the protein was cut from the snapshot. The resulting model contained 7361 atoms. For the QM/MM calculation, the system was partitioned into a QM region that consisted of the Fe-porphyrin ring of the haem (without additional functional groups) and an MM region of all the remaining atoms. The ligand CO and the proximal His120 were also included in the QM region. Along with these essential residues, two neighbouring residues, Arg124 and Leu16, which were implied in experiments to affect the ligand binding, were also included in the QM region. The amino acids were truncated at the $C_{\alpha }\text{–}C_{\beta }$ bond. The QM region in total contained 73 atoms. Atoms within 7 Å of the QM region were relaxed during QM/MM geometry optimizations, while the remaining atoms were frozen. QM/MM calculations were performed by Py-ChemShell invoking NWChem [28] and DL\_POLY [65] for the DFT and molecular mechanics calculations, respectively. Geometries were optimized using the built-in DL-FIND [53] module of Py-ChemShell. The electrostatic embedding scheme with charge shift correction was used to represent the surrounding MM partial charge distribution. The B3LYP density functional [66,67] with the DFT-D3 dispersion correction [73] was used for the QM atoms in all QM/MM calculations. The def2-SVP basis set [68,69], was used for all QM atoms except Fe, which was treated with the def2-TZVP basis set [68,69]. The MM region was described using the CHARMM36 forcefield parameters [62] via DL\_FIELD [74]. We determined Fe to be in its reduced Fe(II) and doublet spin state. Optimizations in other higher spin states resulted in geometries with significantly higher energies, thereby rendering the doublet spin state to be the ground state.

QM/MM optimization was followed by vibrational frequency calculation where a smaller vibrationally active region was defined, consisting of the Fe-porphyrin ring of the haem, the ligand CO and the His120 side chain. Further assignment of IR and Raman signatures is based on vibrations of the active species. In previous experimental work [75], ultraviolet incident light of 405.4 nm wavelength was used for the RR spectra, while the method for determining the value for the calculated spectrum is described below.

### (g) Hybrid QM/MM model for zeolite

**Figure 2.**
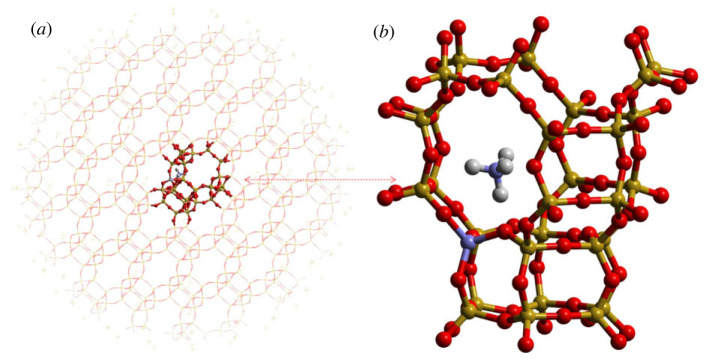
(*a*) QM/MM cluster for the zeolite SSZ-13 structure (QM region in (*b*)): for the zeolite molecular graphics, red is reserved for oxygen, gold for silicon, grey for hydrogen, blue for nitrogen and aluminium (in the ring and on framework, respectively, in the QM region). (Online version in colour.)

For application of the developed methodology to zeolites, the hybrid QM/MM zeolite model developed by Sherwood *et al.* is employed [76–79]. In this model, the Hill and Sauer zeolite forcefield is used for the MM environment [80]. The QM/MM model shown in figure 2 is created by cutting a sphere from a periodic zeolite structure relaxed by the MM forcefield. Outside the QM/MM cluster, point charges are distributed to reproduce accurate electrostatic potential and field due to the extended environment on the inner QM/MM active region.

The QM part, consisting of a chabazite (CHA) cage with an acid site and ammonia, is modelled by DFT methods using the NWChem quantum chemistry package [28] with the def2-TZVP basis set placed on the inner QM region and def2-SVP on its periphery including link atoms [69,81–83], and the hybrid B97-2 exchange-correlation density functional [84,85]. The MM part is modelled using the GULP package [29,86,87]. There are approximately 110 atoms in the QM region and 5900 atoms in the MM region, spanning the whole spherical QM/MM cluster with a radius of 27 Å. For the QM calculations in the presence of point charges, the oxygen-terminated QM region is capped with hydrogen link atoms to satisfy the valency of the terminating oxygen species, with MM charges along the terminating bonds being modified to reproduce correct dipole moments at the regional boundary. Further dipole adjustment is performed to compensate for the spurious replacement of Si-O bonds by O-H linkages at the interface [79]. The embedding potential in this model enters the QM Hamiltonian through point charges located on MM centres and explicitly fitted charges outside the MM region. The MM atoms are divided into two regions: the inner region is allowed to move during the optimization and the outer layer is pre-relaxed on the MM level of theory and held frozen during the QM/MM geometry optimization procedure.

### (h) Hybrid QM/MM model for ZnO surfaces

**Figure 3.**
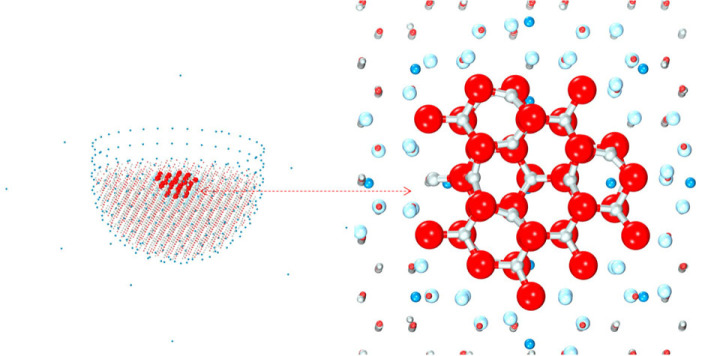
QM/MM cluster for the oxygen-terminated polar ZnO surface. For the ZnO molecular graphics, red is reserved for oxygen, grey for zinc, in the QM region; cyan for the pseudopotential centres; and blue for the fitted point charges. (Online version in colour.)

The final test case focuses on hydrogen chemisorbed on the oxygen-terminated polar surface of ZnO. The stability of this surface has been investigated in detail in [88]. A previously developed hybrid QM/MM model for an active site for hydrogen dissociation [60,89–92] is employed, as illustrated in figure 3. The active site comprises a vacant oxygen interstitial surface site that has originally been proposed to catalyse the syngas to methanol synthetic process [93]. In our early work using a very small QM region, basic hydrogen-related frequencies for this site were successfully assigned [94] partly based on only qualitative arguments about IR and Raman intensities. With the new IR and Raman functionality reported here and the vastly increased power of modern computers, much more realistic large-scale calculations could be performed including the spectral intensities. In this application, QM calculations were performed with NWChem [28] and MM calculations with GULP [29,86,87].

A finite hybrid QM/MM model of the ZnO surface is generated with the active site located at the centre with 61 QM atoms. The model employs a polarizable solid-state embedding scheme in ChemShell developed for ionic systems [90] and uses localizing pseudopotentials centred on cations in the interface region (63 centres) separating the QM atoms from MM environment, comprising over 3200 atoms in the whole model. Parameters of these pseudopotentials have been optimized on the ZnO bulk in [95]. The embedded QM region is described using the B97-2 exchange-correlation density functional; semilocal small-core effective-core potentials [96,97] were placed on zinc QM ions along with a double zeta basis set with polarization functions [98,99]; a triple-zeta basis set with polarization functions (def2-TZVP) [69], with a higher-order polarization f function on oxygen removed, was used for QM hydrogen and oxygen. The remainder of the system was modelled with interatomic potentials [89,91], split into active (15 Å radius) and frozen (12 Å thick) regions, similar to the other QM/MM models described earlier. The fidelity of the electrostatic potential across the active region is guaranteed by point charges outside the whole QM/MM cluster. Hydrogen adsorbate molecules are treated as part of the QM region. The model includes a further oxygen adsorbate occupying one of the nearest vacant oxygen interstitial surface sites, stabilizing two electrons, which simulates the effects of the accumulation layer that is formed in this electron-rich n-type semiconductor.

## 3. Results and discussion

The newly developed features in ChemShell for the calculation of vibrational spectra in realistic environments have been validated for a varied selection of molecular and materials systems using the QM/MM models described in the previous section. The first two test cases concern molecules in solution, namely, the amino acid L-histidine in a spherical water droplet, and a more complex haem protein in a water shell solvation model. The third test case is the heterogeneous catalyst chabazite (CHA), a typical zeolitic material with excellent performance in selective catalytic reduction of nitrogen oxides with ammonia [100–102], which has recently been studied in our group [103,104]. Finally, hydrogen absorption on an ionic metal oxide ZnO polar surface supporting the hydrogenation of carbon dioxide to methanol is investigated.

### (a) Solvated histidine

**Figure 4.**
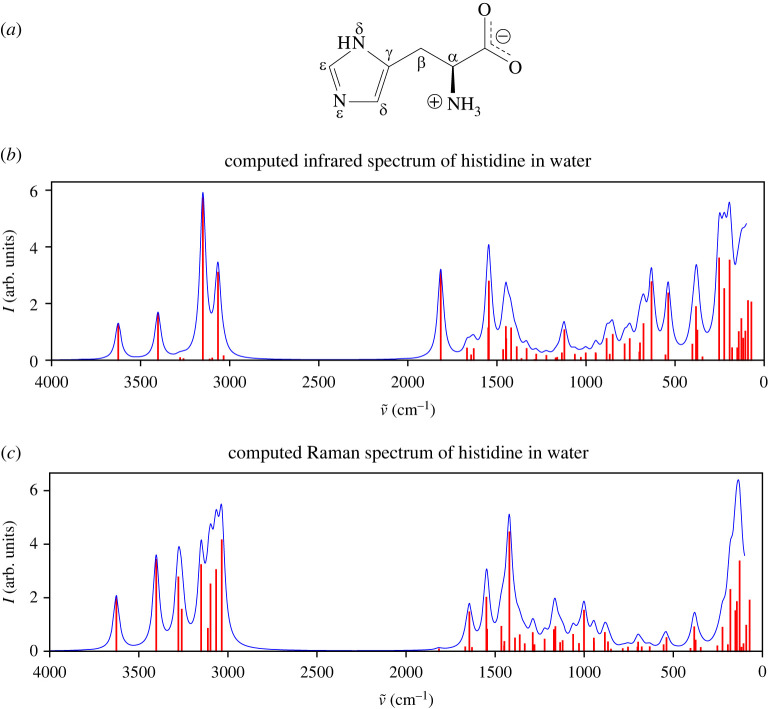
(a) δ-Protonated histidine neutral zwitterion. (b) Computed IR and (c) Raman spectra of δ-protonated histidine in water. Calculated peaks are displayed in red and Lorentzian-broadened bands in blue (bandwidth: $20.0\;{\mathrm{cm}}^{-1}$). (Online version in colour.)

Vibrational signatures in the context of QM/MM modelling were tested first by moving from gas phase to solvated molecules, using the example of a single histidine molecule in explicit water solvent (figure 4). The imidazole side chain of histidine has a nearly neutral $pK_{a}\;(∼6.0)$ [105], and the molecule has several possible protonation and tautomeric states that are sensitive to the chemical environment. Therefore, the IR and Raman spectroscopy of histidine is of special interest for identifying its protonation and tautomeric states and its properties.

The simulated IR spectrum of δ-protonated histidine is shown in figure 4 and the assigned vibrational modes compared with experimental data in table 1. Assignments in the table have been made when one or more computed vibrational modes has, to a significant extent, the same character as those assigned to the experimental data. Visualizations of the assigned modes are given in the electronic supplementary material, section S1.3.1.

**Table 1.** Assignment of vibrational modes of histidine in solution.

vibrational frequencies (${\text{cm}}^{-1}$)			
	experimental	computational	assignment${\;}^{a}$
low-frequency range (2000–1000)			
IR			
	1600	1815	$\nu \;{\left({\mathrm{COO}}^{-}\right)}_{\mathrm{as}}+\delta {\left({\mathrm{NH}}_{3}^{+}\right)}_{\mathrm{as}}$
	1520	1545 (strong)	$\omega ({\mathrm{NH}}_{3}^{+})+\delta (C_{\epsilon }\text{–}H)$
		1549 (weak, appears as shoulder)	$\omega ({\mathrm{NH}}_{3}^{+})+\nu (C_{\epsilon }\text{–}N_{\epsilon })+\delta (C_{\epsilon }\text{–}H)+{\text{H–C}}_{\beta }\text{–H}$ twisting
	1408	1450, 1448	$C_{\beta }\text{–H}\;\mathrm{scissoring}+\nu {\left({\mathrm{COO}}^{-}\right)}_{s}+\nu (C_{\alpha }\text{–}{\mathrm{COO}}^{-})+\delta (N_{\delta }\text{–H})$
		1421	$\nu \;\mathrm{ring}+C_{\beta }\text{–H}\;\mathrm{scissoring}+\delta (N_{\delta }\text{–H})$
	1286, 1088	1121	$\delta (N_{\delta }\text{–H})+\delta (C_{\delta }\text{–H})+\delta (C_{\epsilon }\text{–H})+\nu (N_{\delta }{\text{–C}}_{\epsilon })$
Raman			
	1632	1645	$\nu (C_{\epsilon }\text{–}N_{\epsilon })+\nu (C_{\gamma }\text{–}C_{\delta })+\delta (N_{\delta }\text{–}H)+\delta (C_{\delta }\text{–}H)$
	1575, 1357	1549 (strong)	$\omega ({\mathrm{NH}}_{3}^{+})+\nu (C_{\epsilon }\text{–}N_{\epsilon })+\nu (C_{\gamma }\text{–}N_{\delta })+\delta (C_{\epsilon }\text{–}H)+{\text{H–C}}_{\beta }\text{–H}\;\mathrm{twisting}$
		1545 (weak)	$\omega ({\mathrm{NH}}_{3}^{+})+\delta (C_{\epsilon }\text{–H})$
	1494	1465	$C_{\beta }\text{–H}\;\mathrm{scissoring}+\delta (N_{\delta }\text{–H})$
	1285	1421	$\nu \;\mathrm{ring}+C_{\beta }\text{–}H\;\mathrm{scissoring}+\delta (N_{\delta }\text{–H})$
high-frequency range (3700–3000)			
IR			
		3066, 3150	$\nu {\left({\mathrm{NH}}_{3}^{+}\right)}_{\mathrm{as}}$

${\;}^{a}$Symbols associated with assignments: ν defines stretching, δ defines bending and ω defines wagging. Subscript s stands for the symmetric mode and as for the asymmetric mode.

In the range $2000\text{–}1000\;{\mathrm{cm}}^{-1}$, the strongest calculated absorption peaks are at $1815\;{\mathrm{cm}}^{-1}$ and $1545\;{\mathrm{cm}}^{-1}$, the latter accompanied by a less strong shoulder at $1549\;{\mathrm{cm}}^{-1}$. The peaks at $1545\text{–}1549\;{\mathrm{cm}}^{-1}$ predominantly involve an ${\mathrm{NH}}_{3}^{+}$ wagging mode in combination with $C_{\epsilon }{\text{–N}}_{\epsilon }$, $C_{\epsilon }\text{–H}$ and ${\text{H–C}}_{\beta }\text{–H}\;\mathrm{motions}$, and correspond well with the experimentally assigned band with imidazole ring stretch and NH${\;}_{3}^{+}$ symmetric bend character at $1520\;{\mathrm{cm}}^{-1}$.

The peak at $1815\;{\mathrm{cm}}^{-1}$ is a combination of a ${\mathrm{COO}}^{-}$ asymmetric stretch and ${\mathrm{NH}}_{3}^{+}$ bend. It is of similar character but substantially shifted from the experimentally assigned ${\mathrm{COO}}^{-}$ asymmetric stretch at $1600\;{\mathrm{cm}}^{-1}$. As other experimental studies show no significant IR activity between 1750 and $2250\;{\mathrm{cm}}^{-1}$ [106], it is unlikely to correspond to any other experimental peak.

Further smaller but significant signals are found in the range of $1420\text{–}1450\;{\mathrm{cm}}^{-1}$, where the simulated modes again agree well with experimental data. They are primarily contributed by C–H scissoring at $C_{\beta }$ ($1421\;{\mathrm{cm}}^{-1}$) and ${\mathrm{COO}}^{-}$ symmetric stretching with a $C_{\alpha }{\text{–COO}}^{-}$ stretch at $1450\;{\mathrm{cm}}^{-1}$, both coupled by an $N_{\delta }\text{–H}\;\mathrm{bend}$. This corresponds with an experimentally observed symmetric ${\mathrm{COO}}^{-}$ stretch at $1408\;{\mathrm{cm}}^{-1}$.

The medium sized peak at $1121\;{\mathrm{cm}}^{-1}$ is due to the bending of N${\;}_{\delta }$–H, $C_{\delta }\text{–H}$ and $C_{\epsilon }\text{–H}$, as well as an $N_{\delta }{\text{–C}}_{\epsilon }$ stretch in the imidazole ring. This result corresponds to a weak experimental $N_{\delta }{\text{–C}}_{\epsilon }$ stretch and $C_{\epsilon }\text{–H}\;\mathrm{bend}$ at $1286\;{\mathrm{cm}}^{-1}$, and $N_{\delta }{\text{–C}}_{\epsilon }$ stretch and $C_{\delta /\epsilon }\text{–H}\;\mathrm{bend}$ at $1088\;{\mathrm{cm}}^{-1}$ [107].

In the high-frequency range above $3000\;{\mathrm{cm}}^{-1}$, the calculated spectrum is dominated by an asymmetric N–H stretch of the ${\mathrm{NH}}_{3}^{+}$ group at 3066 and $3150\;{\mathrm{cm}}^{-1}$.

Overall, our calculations qualitatively reproduce the experimentally observed IR spectra of histidine in the aqueous solution at pH 7, with the exception of the $1815\;{\mathrm{cm}}^{-1}$ peak. It should be pointed out that the current example is based only on a certain tautomer without considering other possibly coexisting species of various protonation states in reality, and so may not capture the full experimental vibrational signatures. Moreover, only a single snapshot, namely, a conformer, has been calculated, and using multiple snapshots could improve the results. However, the primary purpose of the calculation is to demonstrate the capability of our implementation, while a future systematic study would need to take into account multiple molecular dynamics snapshots, tautomers, various protonation states and hydrogen bonding effects. Another possible cause of discrepancy is missing anharmonic contributions, which will be addressed in the future development work.

The computed Raman spectrum is shown in figure 4 and assigned Raman vibrational frequencies in table 1. Again, the assignments in the range $2000\text{–}1000\;{\mathrm{cm}}^{-1}$ generally agree with experiment. A relatively less strong calculated peak at $1645\;{\mathrm{cm}}^{-1}$ is characterized as a $C_{\epsilon }{\text{–N}}_{\epsilon }$ stretch, together with a $C_{\gamma }{\text{–C}}_{\delta }$ stretch and $C_{\delta }\text{–H}\;\mathrm{bend}$ with $N_{\delta }\text{–H}\;\mathrm{bend}$ in the ring, fully in line with the experimental literature assignment of the weakened $1632\;{\mathrm{cm}}^{-1}$ peak in the neutral solution [107].

There is a stronger peak at $1549\;{\mathrm{cm}}^{-1}$ formed by the $C_{3v}\text{-symmetric}$ wagging of ${\mathrm{NH}}_{3}^{+}$, $C_{\epsilon }{\text{–N}}_{\epsilon }$ stretch, $C_{\gamma }{\text{–C}}_{\delta }$ stretch, $C_{\epsilon }\text{–H}\;\mathrm{bend}$ and a ${\text{H–C}}_{\beta }\text{–H}\;\mathrm{twist}$, which is the same normal mode at the same position in the IR spectrum. The peak is strengthened by a closely positioned weaker signal at $1545\;{\mathrm{cm}}^{-1}$, also overlapping the position in the IR spectrum, albeit with reversed strengths. The two quasi-coinciding modes could match the characteristic strong $1575\;{\mathrm{cm}}^{-1}$ band observed in the experiment, which is however assigned primarily to $C_{\gamma }{\text{–C}}_{\delta }$. The experimental $1357\;{\mathrm{cm}}^{-1}$ band is also reported to feature a $C_{\epsilon }{\text{–N}}_{\epsilon }$ stretch and $C_{\gamma }{\text{–C}}_{\delta }$ stretch, but is not suggested to have a contribution from ${\mathrm{NH}}_{3}^{+}$ [107].

${\text{H–C}}_{\beta }\text{–H}$ scissoring and an $N_{\delta }\text{–H}\;\mathrm{bend}$ forms a shoulder at $1465\;{\mathrm{cm}}^{-1}$, partially supporting the assignment to the experimental $1494\;{\mathrm{cm}}^{-1}$ band, which is suggested to be principally an $N_{\delta }\text{–H}\;\mathrm{bend}$ [107]. Finally, a significant peak is found by our calculation at $1421\;{\mathrm{cm}}^{-1}$ that arises from various ring bond stretches, an $N_{\delta }\text{–H}\;\mathrm{bend}$ and scissoring of ${\text{H–C}}_{\beta }\text{–H}$. This rather complex mode could be associated with the strong experimental peak at $1286\;{\mathrm{cm}}^{-1}$, although significantly shifted in frequency, which is reported to be an $N_{\delta }{\text{–C}}_{\epsilon }$ bond stretch in the imidazole ring [107].

Also, our results in general compare well with DFT calculations with an explicit water cluster plus the continuum solvent model in predicting the IR and Raman spectra made by Deplazes *et al.* [106]. In their study, the strong infrared signatures of ${\mathrm{COO}}^{-}$ and ${\mathrm{NH}}_{3}^{+}$ were calculated to be 1610 and $1646\;{\mathrm{cm}}^{-1}$, respectively. Again, this discrepancy is likely to be caused by single conformation used in our demonstrative calculation. In the computed Raman spectrum, both methods predict similar patterns for the series of significant peaks spanning from 1200 to $1700\;{\mathrm{cm}}^{-1}$.

### (b) Vibrational spectroscopy of the CO-bound haem protein *Ax*CYTcp

Resonance Raman spectroscopy is extensively used to extract structural information of the haem group in haem-containing proteins. The high-frequency porphyrin skeletal modes that are Raman active are very sensitive to ligation, redox and spin states of the haem, and hence, RR spectra can provide detailed information of the haem site for a particular protein [108]. With the advancement of computational resources, simulation of RR spectra provides a cheaper and less-time consuming alternative to obtain information about the *in situ* haem environment within a protein, and any alteration to it that could be caused by changes in ligation, redox, mutation or other factors. As a case study to simulate RR spectra of proteins in solution, we have studied cytochrome c’ from *Alcaligenes xylosoxidans* (*Ax*CYTcp), which is present in denitrifying bacteria. These are penta-coordinated monohaem proteins that can discriminate small gaseous ligands like NO and CO by binding to opposite faces of haem, while molecular oxygen is not bound [109,110]. In *Ax*CYTcp, NO binds to the proximal side of the haem, resulting in a final penta-coordinate ligand complex, whereas CO binds to the distal side of the haem and yields a hexa-coordinated ligand complex (6cCO). Experimental RR studies are available for AxCYTcp in both solution and crystalline states [70,75]. In this study, we simulate the RR spectrum for the 6cCO protein solvated in water, and compare the calculated Raman frequencies with high-frequency porphyrin skeletal signatures from solution and solid-state studies.

**Figure 5.**
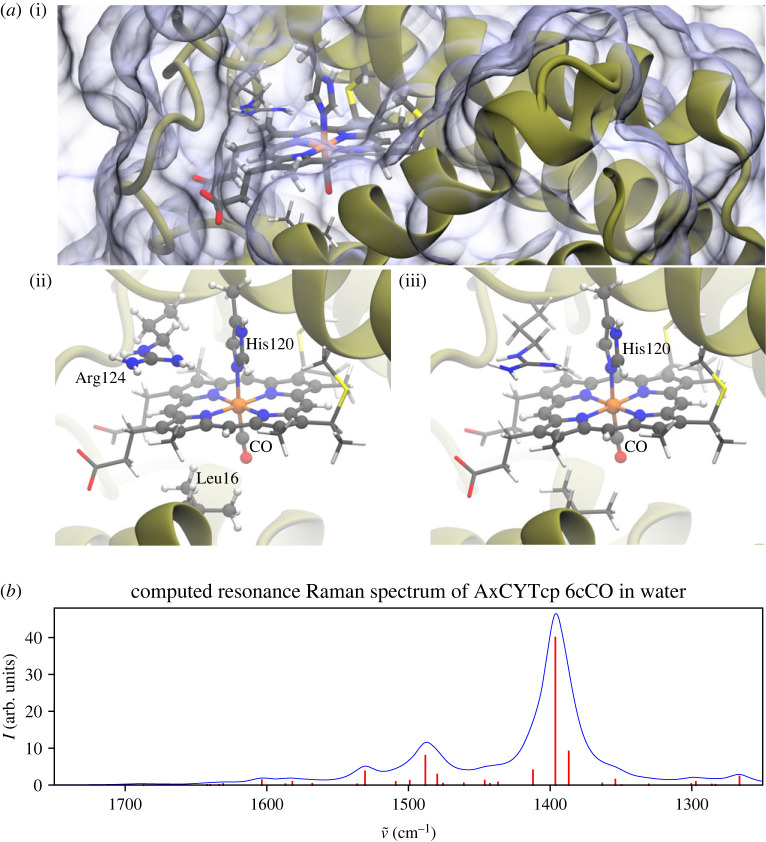
(a) (i) QM/MM embedded 6cCO *Ax*CYTcp (six-coordinate CO bound) protein in aqueous solution; (ii) the QM region in the enzymatic active site (ball-and-stick) and (iii) the vibrationally active region in the spectroscopic calculations (ball-and-stick). (b) Computed RR spectrum of 6cCO *Ax*CYTcp. Calculated peaks are displayed in red and Lorentzian-broadened bands in blue (bandwidth: $10.0\;{\mathrm{cm}}^{-1}$). (Online version in colour.)

As shown in figure 5, in the centre of the enzyme’s active site, Fe is bound to a CO molecule on the distal face and His120 in the proximal face, to form a six-coordinate structure. After carrying out full geometry optimizations with various multiplicities, we have confirmed that the doublet spin state of reduced Fe(II) is the ground state with the lowest energy.

To calculate the RR spectrum of the solvated 6cCO haem protein structure, we first calculated the electronic excited states of the system using TDDFT [58] and then analysed the associated transitions (see the electronic supplementary material for more details). The 14th excitation, at a wavelength of 374 nm, corresponds to the most probable electronic transition, with the second most probable transition coming from the 13th root at 376 nm. These transitions were identified as the relevant Soret band excitations based on their strength and the character of the molecular orbitals involved. The 14th excitation was therefore used for the calculation of the resonance polarizabilities with NWChem, [12,56,58,59] which were in turn used by Py-ChemShell to calculate the RR intensities.

The calculated and assigned Raman vibrational modes are presented in table 2. The calculated normal modes are delocalized over the whole porphyrin, with coupled stretching and bending modes. In addition, there are also contributions from the coordinated histidine residue. To assign the modes, we compared the character of the porphyrin modes with the experimental band assignments, though in many cases there are significant additional contributions from porphyrin motions that are not assigned to a specific description. Visualization of the vibrational modes highlighting the motions used for the assignments are in the electronic supplementary material, section S1.4.2.

**Table 2.** Assignment of Raman vibrational frequencies for 6cCO *Ax*CYTcp.

Raman vibrational frequencies (${\text{cm}}^{-1}$)			
experimental	computational	assignment${\;}^{a}$	porphyrin local coordinate${\;}^{a}$ [111]
high-frequency range (1700–1300)			
1634${\;}^{b}$ (very weak)	negligible	$\nu_{10}$	$\nu {\left(C_{\alpha }{\text{–C}}_{m}\right)}_{\mathrm{as}}$
1597${\;}^{b}$, 1598${\;}^{c}$	1582, 1587, 1603	$\nu_{2}$	$\nu (C_{\beta }{\text{–C}}_{\beta })$
1370${\;}^{b}$, 1369${\;}^{c}$ (strong)	1396	$\nu_{4}$	$\nu {\left(\text{Pyr, half-ring}\right)}_{s}$
low-frequency range (700–500)			
482, 493 [100K]${\;}^{b}$,	483, 492	ν(Fe−CO)	—
491 [RT]${\;}^{b}$			
570, 580 [100K]${\;}^{b}$,	600, 608	δ(Fe−CO)	—
572 [RT]${\;}^{b}$			

${\;}^{a}$Symbols associated with assignments: ν defines stretching and δ defines bending. Subscript s stands for symmetric mode and as for asymmetric mode.

${\;}^{b}$Measurement in solution [70].

${\;}^{c}$Measurement in protein crystal [75].

RR experiments in single crystals and solution have shown porphyrin marker bands in the region of $1300\text{–}1700\;{\mathrm{cm}}^{-1}$ [70,75]. The observed intense peak at $1370\;{\mathrm{cm}}^{-1}$ in both phases is assigned to the pyrrole half-ring symmetric stretch, which is conventionally referred to as $\nu_{4}$ in terms of porphyrin local coordinates [112]. Our calculation for this mode is in good agreement with a peak at $1396\;{\mathrm{cm}}^{-1}$, with the expected strong intensity; it also agrees with an earlier spectral study of ferrous yeast iso-1 cytochrome *c* and its isotopomers [113].

In the crystalline state, a weak band at $1598\;{\mathrm{cm}}^{-1}$ was reported and assigned as $\nu_{2}$, which corresponds to the $C_{\beta }{\text{–C}}_{\beta }$ stretching mode [75]. In solution, $\nu_{2}$ was observed at almost the same frequency ($1597\;{\mathrm{cm}}^{-1}$). Our simulated solution spectrum shows this band with contributions from peaks at $1582\;{\mathrm{cm}}^{-1}$, $1587\;{\mathrm{cm}}^{-1}$ and $1603\;{\mathrm{cm}}^{-1}$, all similarly with $\nu_{2}$ character. The relative intensity is considerably weaker than the $\nu_{4}$ peak, as expected.

In experimental solution data, an additional very weak signal was reported with $\nu_{10}$ character at $1634\;{\mathrm{cm}}^{-1}$, which corresponds to an asymmetric $C_{\alpha }{\text{–C}}_{m}$ stretch. In the simulated spectrum, there is a peak also at $1634\;{\mathrm{cm}}^{-1}$ with the expected $\nu_{10}$ character, but the calculation intensity is negligible, and so this region of the simulated spectrum more closely resembles experimental measurements on the protein crystal, where no significant $\nu_{10}$ peak is observed. In the simulated spectrum, a more pronounced peak is observed at $1488\;{\mathrm{cm}}^{-1}$, with $\nu_{3}$ character, corresponding to a symmetric $C_{\alpha }{\text{–C}}_{m}$ stretch. In both the experimental crystal and solution spectra, small peaks can be seen in this region, assigned to $\nu_{3}$ in the solution case, but compared with the calculated spectrum they are much weaker.

CO stretching and bending modes appear in the low-frequency region ($400\text{–}600\;{\mathrm{cm}}^{-1}$). Fe–CO stretching modes are calculated at 483 and $492\;{\mathrm{cm}}^{-1}$, fully agreeing with the experimentally observed 493 and $482\;{\mathrm{cm}}^{-1}$ (at 100 K) and $491\;{\mathrm{cm}}^{-1}$ (at room temperature) [70]. The Fe–CO bending mode is calculated to be at 608 and $600\;{\mathrm{cm}}^{-1}$, which also corresponds well with the experimental assignment at 570 and $580\;{\mathrm{cm}}^{-1}$ (at 100 K) and $572\;{\mathrm{cm}}^{-1}$ (at room temperature) [70]. Visualizations of the identified vibrational modes in the high- and low-frequency ranges are given in the Supporting Information.

In summary, the computed RR results overall reproduce the reported experimental RR spectroscopy measurements at a good level of agreement, giving a spectrum and modes characteristic of a six-coordinated CO-bound *Ax*CYTcp system, where a single strong peak is observed with $\nu_{4}$ character. They also demonstrate that our implementation of the IR/Raman method in the QM/MM context is capable of helping to resolve RR spectra of highly intricate chemical structures such as enzymes.

### (c) Identification of ammonia species in Cu-containing zeolite SSZ-13

In this section, computational IR and Raman spectra obtained from the QM/MM calculation are used to identify the ammonia species in CHA structured zeolite SSZ-13, where they can adsorb on framework Brønsted acid and extraframework copper ion sites, which are active sites in ammonia selective catalytic reduction of nitrogen oxides (${\text{NH}}_{3}\text{-SCR}$) [103,114].

Zeolites are nanoporous silicate materials, in which aliovalent cations can occupy tetrahedral framework and extraframework sites giving rise to chemical activity. As reported in previous experimental work using FTIR spectroscopy [114–116], protonation of ammonia is a dominant process in acidic zeolite environments at relatively low temperatures, while at high temperatures, ammonium ${\text{NH}}_{4}^{+}$ species are more likely to decompose gradually into ${\text{NH}}_{3}$ and ${\text{H}}^{+}$, along with release of other unwanted combustible ${\text{NO}}_{x}/{\text{N}}_{2}\text{O}$ gases. By contrast, with slight elevation of temperature, the release on heating of dissociated ${\text{NH}}_{3}$ species from ${\text{Cu}}^{2+}$ Lewis acid sites in the SSZ-13 structures occurs at a much higher rate [114]. Therefore, detection of ammonia species associated with two types of acid sites is an important problem in the material’s characterization.

**Figure 6.**
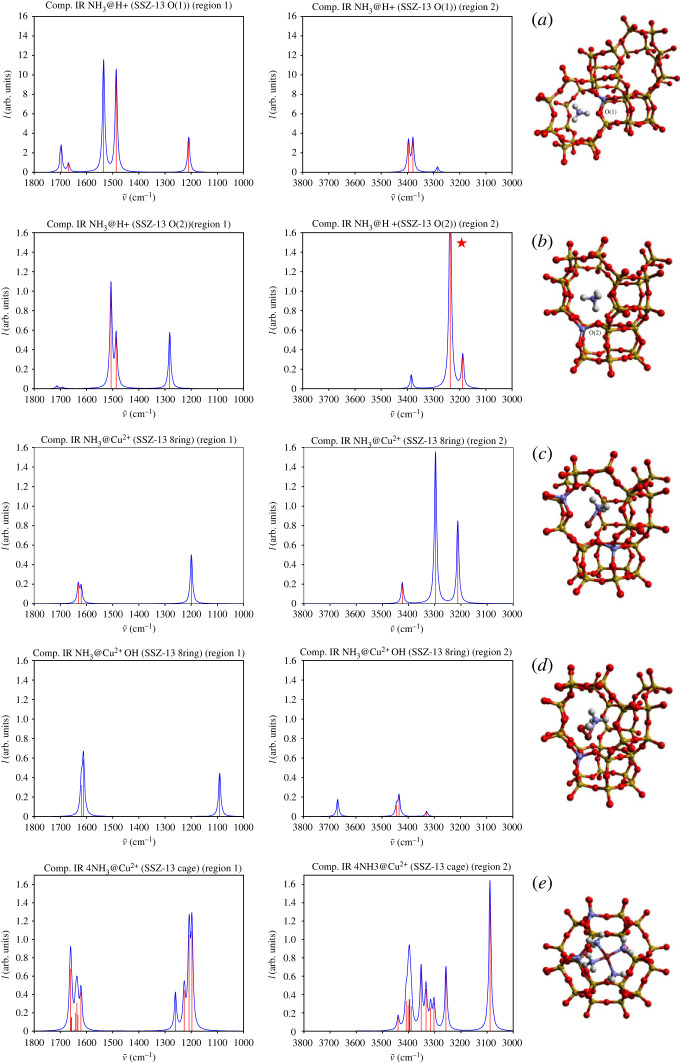
Computational IR spectra in two regions ($1000\text{–}1900\;{\text{cm}}^{-1}$ and $3000\text{–}3800\;{\text{cm}}^{-1}$) capturing signatures from wagging and bending motions of ${\text{NH}}_{3}$ species adsorbed at different acid sites. (*a*) ${\text{NH}}_{3}$ absorption at O(1) Brønsted acid site (junction of eight- and four-member rings) forming stable ammonium. (*b*) Ammonia absorption at O(2) Brønsted acidic site (junction of eight-, six- and four-member rings) forming stable ammonium. (*c*) Adsorption of ${\text{NH}}_{3}$ at ${\text{Cu}}^{2+}$ site in the eight-member ring with hydrogen and ${\text{Cu}}^{2+}$ interacting with the framework oxygen. (*d*) ${\text{NH}}_{3}$ adsorption at hydroxylated ${\text{Cu}}^{2+}$ site in low-aluminium density SSZ-13. (*e*) Adsorption of ${\left({\text{NH}}_{3}\right)}_{4}$ complexes at ${\text{Cu}}^{2+}$ ion stabilized in the cage. Only QM regions containing chemical active sites are shown.

IR spectroscopy was used in a previous study to identify the ${\text{NH}}_{3}$ species adsorbed on the ${\text{Cu}}^{2+}$ extraframework sites [114]. Four Brønsted acid sites have been detected in CHA, but only two sites, O(1) and O(2), are typically involved in the ${\text{NH}}_{3}$-SCR process [114] and therefore are investigated in this study. The O(1) site is at the junction of eight- and four-member rings, whereas O(2) is at the junction of eight-, six- and four-member rings. The structure of ammonia adsorbed on these two sites is shown in figure 6*a*,*b*. A ${\text{Cu}}^{2+}$ ion in zeolites can compensate either one or two framework aluminium sites. In low-aluminium density zeolites, it would be charge-neutralized by an ${\text{OH}}^{-}$ group. The optimized structure includes two bonds between copper and framework oxygen sites coordinated to aluminium, i.e. one O(1) and one O(2), as shown in figure 6*c*. In the high-aluminium density zeolite, there is a possibility of two aluminium species appearing in the same eight-member ring [117–119], and this is the case that has been chosen for investigation in this article. Satisfying both the Löwenstein and Dempsey rules [120,121], aluminium ions are not expected to occupy nearest and next nearest T-sites of the framework. Considering the closest aluminium separation across an eight-member ring that has been reported as a site for copper location in SSZ-13 [117–119], the plausible di-aluminium structure with ${\text{Cu}}^{2+}$ in the middle is theoretically predicted as shown in figure 6*d*. In this configuration, the copper ion is seen to coordinate to three framework oxygen sites including two O(1) sites and one O(2) site.

Adsorption of ${\text{NH}}_{3}$ has been modelled on the four selected active sites with resultant structures shown in figure 6*a*–*d*. The optimized structures of Brønsted acid sites with ammonium adsorbate are shown in figure 6*a*,*b*. The ${\text{NH}}_{3}$ molecule was initially put in the middle of a zeolite cage, following which a proton transfer is observed, forming stable ammonium at O(1) and O(2) sites. This finding agrees with experimental reports that ammonia reduces acidic sites in zeolites at low temperatures [115]. The low- and high-frequency IR signatures at $1210\;{\text{cm}}^{-1}$ and $1282\;{\text{cm}}^{-1}$ represent wagging motions of the newly formed ${\text{NH}}_{4}^{+}$ at O(1) and O(2) sites, respectively. The O(2) site is characterized by signature of higher intensity coming from the wagging motion. As detailed in table 3, bending motions of the ${\text{NH}}_{4}^{+}$ species are observed at $1486\;{\text{cm}}^{-1}$ and $1534\;{\text{cm}}^{-1}$ for symmetrical and $1669\;{\text{cm}}^{-1}$, $1697\;{\text{cm}}^{-1}$ for antisymmetrical scissoring motions at O(1). $1487\;{\text{cm}}^{-1}$, $1506\;{\text{cm}}^{-1}$, $1691\;{\text{cm}}^{-1}$ and $1713\;{\text{cm}}^{-1}$ peaks account for similar bending and scissoring motions at O(2). Both symmetrical and scissoring vibrations have distinct orientations (see the electronic supplementary material for the vectors of normal modes). The two antisymmetrical scissoring motions at the O(2) site are uniformly blue shifted in the IR spectra compared with the counterparts at O(1), but with much weaker signals (figure 6*b* at $1713\;{\text{cm}}^{-1}$ region). Therefore, four symmetrical bending motions from different Brønsted sites are assigned, and together they are the major contributions to the bending region in the IR spectra discussed in [114] with high shoulders attributed to bending modes in a $1300\text{–}1600\;{\text{cm}}^{-1}$ domain.

**Table 3.** Assignment of vibrational modes of ammonia adsorbates in zeolite SSZ-13.

frequencies of adsorbed species (${\text{cm}}^{-1}$)				
		computational		
${\text{NH}}_{4}^{+}$@Brønsted	experimental	O(1)	O(2)	assignment
range: 1800–1200				
	1278 (IR) [114]	1209.52	1282.11	$\omega ({\text{NH}}_{4}^{+})$
	1393–1401 (IR) [114]	1486.49	1486.53	$\delta_{\text{s}}({\text{NH}}_{4}^{+})$
	1455–1448 (IR) [114]	1534.49	1506.35	$\delta_{\text{s}}({\text{NH}}_{4}^{+})$
	—	1668.99	1691.34	$\delta_{\text{as}}({\text{NH}}_{4}^{+})$
	—	1696.82	1712.61	$\delta_{\text{as}}({\text{NH}}_{4}^{+})$
range: 3800–3000				
	3400–3100 (IR) [114]	3284.90	3188.64	$\nu ({\text{NH}}_{4}^{+})$
	3400–3100 (IR) [114]	3378.90	3236.35	$\nu ({\text{NH}}_{4}^{+})$
	3400–3100 (IR) [114]	3395.88	3385.12	$\nu ({\text{NH}}_{4}^{+})$
${\text{NH}}_{3}$@${\text{Cu}}^{2+}$		lone	hydroxyl.	
range: 1800–1000				
	—	1199.47	1091.37	$\omega ({\text{NH}}_{3})$
	1619 (IR) [114]	1620.19	1611.59	$\delta ({\text{NH}}_{3})$
	1619 (IR) [114]	1631.46	1619.77	$\delta ({\text{NH}}_{3})$
range 3800–3000				
	3400–3100 (IR) [114]	3211.75	3329.40	$\nu ({\text{NH}}_{3})$
	3400–3100 (IR) [114]	3297.05	3434.88	$\nu ({\text{NH}}_{3})$
	3400–3100 (IR) [114]	3424.02	3444.66	$\nu ({\text{NH}}_{3})$
	—	—	3669.64	ν(OH)
${\left({\text{NH}}_{3}\right)}_{4}$@${\text{Cu}}^{2+}$		lone		
range 1800–1000				
	1278 (IR) [114]	1197.34–1260.31		$\omega ({\left({\text{NH}}_{3}\right)}_{4})$
	1620 (IR) [114]	1621.01–1661.53		$\delta ({\left({\text{NH}}_{3}\right)}_{4})$
range 3800–3000				
	3400–3100 (IR) [114]	3088.74–3439.98		$\nu ({\left({\text{NH}}_{3}\right)}_{4})$

Turning our attention to ammonia adsorbates on copper Lewis sites, distinct behaviour is observed in the vibrational IR intensities for ${\text{Cu}}^{2+}$ sites in low- and high-aluminium density environments (figure 6*c*,*d*). The frequencies of wagging motions of the ${\text{NH}}_{3}$ adsorbate at ${\text{Cu}}^{2+}$, shown in figure 6*d*, have been red shifted to $1091\;{\text{cm}}^{-1}$ by the presence of the neighbouring hydroxyl group, when compared with the adsorption at a lone ${\text{Cu}}^{2+}$ ion. The IR intensities of bending motions at 1612 and $1620\;{\text{cm}}^{-1}$ are found to be stronger compared with the ${\text{NH}}_{3}$ species adsorbed on the ${\text{Cu}}^{2+}$ in figure 6*c*. The band position agrees with experimental IR signatures at $1620\;{\text{cm}}^{-1}$ assigned for weakly adsorbed ${\text{NH}}_{3}$ at the Lewis site [114].

The frequencies of the wagging motion differ significantly from ammonium adsorbed on the O(2) site to ammonia stabilized on the ${\text{Cu}}^{2+}$ centre ($\Delta \nu =191.11\;{\text{cm}}^{-1}$), see figure 6*b*,*d*. The difference in wagging frequencies for the ammonium ions at O(1) and O(2) sites proves to be much lower ($\Delta \omega =73\;{\text{cm}}^{-1}$), but the interaction with different framework sites strongly affects the IR intensities (cf. figure 6*a*,*b*). On interaction with OH, a red shift is observed of similar magnitude, see figure 6*c*,*d*, whereas interaction of ammonium with O(2) site leads to a blue shift and a higher intensity, comparing with O(1) site.

Under working conditions, higher loading of ammonia per copper site in SSZ-13 is typically used [102,114]. To test the effect of ammonia loading, a ${\left[\text{Cu}{\left({\text{NH}}_{3}\right)}_{4}\right]}^{2+}$ complex was next optimized in the CHA cage and associated IR signatures calculated, with results shown in figure 6*e*. The ${\text{Cu}}^{2+}$ ion is coordinated with the four ${\text{NH}}_{3}$ molecules forming a planar structure with hydrogen interacting with framework oxygen. Wagging and bending motions appear to be delocalized over pairs of ${\text{NH}}_{3}$ species that are observed with different orientations and amplitudes for different signature motions. Wagging is seen in the $1200\text{–}1300\;{\text{cm}}^{-1}$ spectral region, whereas bending motions concentrate in the 1620–1660 region, showing good agreement with experimental findings. Among these collective motions, high-intensity signatures are associated with interactions of hydrogen species with the framework oxygen.

The stretching motions of N-H bonds in the ammonium ion show significant interactions between protons and framework oxygen, with hydrogen bond lengths of 1.464 Å and 1.635 Å at O(1) and O(2), respectively, whereas no such strong interactions are observed for the ammonia adsorbates. Calculated signatures of stretching modes in the region above $3000\;{\text{cm}}^{-1}$ (figure 6*a*,*b*) show good agreement with experimental reports [114,115]. On interaction with the framework, the calculated signature at $3236\;{\text{cm}}^{-1}$ in figure 6*b*, labelled with a star, has significantly stronger intensity compared with other peaks and has been truncated to allow the other peaks to be seen clearly. Importantly, hydroxylation of the ${\text{Cu}}^{2+}$ results in lower N-H symmetrical and asymmetrical stretching modes at 3329, 3435 and $3445\;{\text{cm}}^{-1}$ with significantly reduced intensity (figure 6*c*,*d*). A detailed investigation of vibrational behaviour of physi- and chemisorbed ammonia over CHA will be reported elsewhere [122].

**Figure 7.**
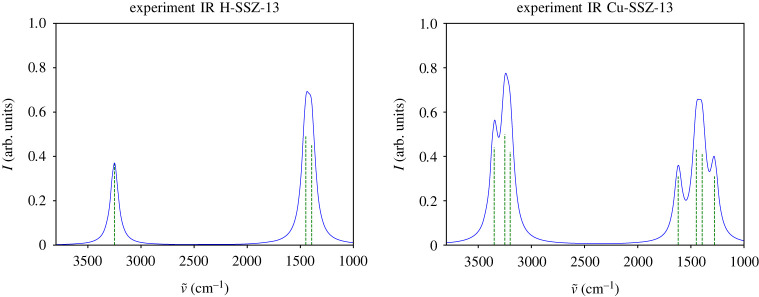
Experimental IR spectra of H-SSZ-13 and Cu-containing SSZ-13 zeolites, based on fig. 4 in [114].

The work presented in this section is summarized in table 3 with selected band assignments relating theory and experiment for the ${\text{NH}}_{3}$ adsorbates at different acid sites. The calculated IR intensities compare well with experimental results [114]. Experimentally observed IR peaks centring at ca. $1400\;{\text{cm}}^{-1}$ are shifted to a somewhat higher wave number in our calculations and feature ${\text{NH}}_{4}^{+}$ vibrations shown in figure 6*a*,*b*. Further to the experimental analysis [114], at least eight bending motions of ${\text{NH}}_{4}^{+}$ species at the two different Brønsted sites can be seen contributing to these signatures (1486.49–$1712.61\;{\text{cm}}^{-1}$ in table 3). The relative computational intensities of these peaks, compared with those at ca. $1282.11\;{\text{cm}}^{-1}$ and those from 1611.59 to $1631.46\;{\text{cm}}^{-1}$ (figure 6*c*,*d*), agree well with the experiment (figure 7).

### (d) Evolution of hydrogen signatures on oxygen-terminated ZnO polar surface

Zinc oxide is widely used as a catalyst and support in synthesis of bulk chemicals, an important example of which is the production of methanol from syngas [93,123]. Hydrogenation of ZnO surfaces is a key stage of the catalytic process. In this section, hydrogen chemisorbed on ZnO oxygen-terminated polar surfaces is investigated using IR and Raman calculations in Py-ChemShell employing the hybrid QM/MM approach with polarizable embedding.

Previous experimental and computational studies of hydrogen adsorption on ZnO surfaces concluded that hydrogen motion at oxygen and zinc vacant surface interstitial sites is the source of observed vibrational signatures, including a zinc hydride stretching mode at $1710\;{\text{cm}}^{-1}$ [94,124,125]. Of particular interest are emerging IR and Raman signatures around 1600–$1710\;{\text{cm}}^{-1}$, which evolve on exposure of pristine ZnO surfaces to hydrogen [125,126]. These spectral features were attributed to hydrogen adsorbed at surface oxygen vacant sites as hydride [94]. On increasing hydrogen adsorption, signatures of interest intensify in both IR and Raman spectra.

We first performed calculations on the pristine ZnO polar surface following the experimental procedure [124]. There were no computational IR or Raman spectral signals beyond $1000\;{\text{cm}}^{-1}$, in line with the experiment. All spectral features only come from lower-frequency surface phonon modes.

**Figure 8.**
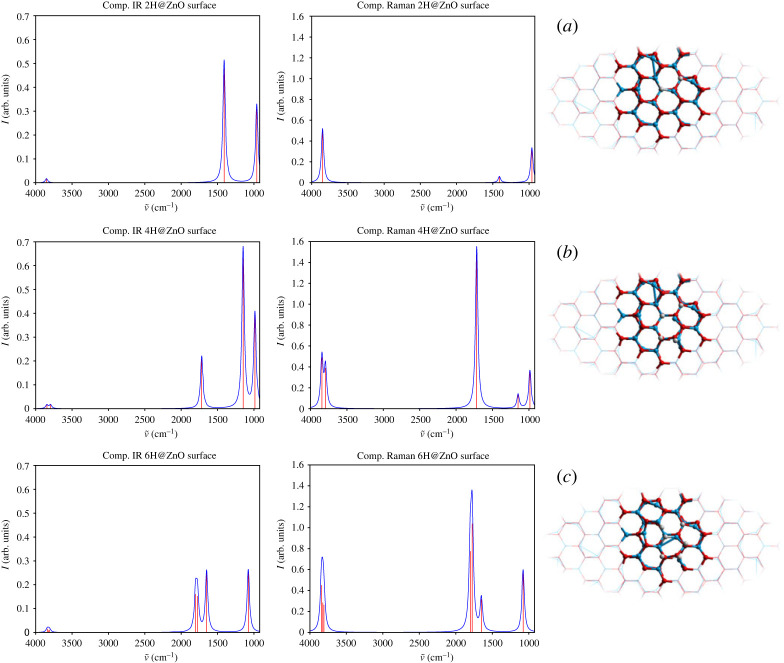
Computational infrared and Raman spectra of the oxygen-terminated ZnO polar surfaces with adsorption of hydrogen species, comparing the dissociation of (*a*) one hydrogen molecule, (*b*) two hydrogen molecules and (*c*) three hydrogen molecules.

On adsorption, new spectral signatures appear as hydrogen molecules dissociate on the surface heterolytically, giving rise to a proton and a hydride species coordinated to surface oxygen and zinc, respectively (as shown in figure 8*a*). Geometry optimization of the QM/MM model with one hydrogen molecule results in a hydroxyl group oriented normal to the surface with a bond length of 0.960 Å, whereas the hydride is found within the nearest surface oxygen vacant site bridging two coordinated Zn atoms (with bond lengths of 1.708 Å and 1.748 Å) and at 2.263 Å from the third undercoordinated zinc site in the vacancy. The formation of proton and hydride species is confirmed by Mulliken analysis of charge densities. The calculated adsorption energy of 1.56 eV confirms that the process is highly exothermic and therefore irreversible (type-II, which we identify here as due to hydride bridging configuration) [124,125]. The calculated signal at $1408\;{\text{cm}}^{-1}$ is due to an asymmetric stretching mode of the Zn-H-Zn hydride bridging species (figure 8*a* and the electronic supplementary material). The symmetric Zn-H-Zn stretching mode appears at $960\;{\text{cm}}^{-1}$. In the high-frequency spectral region, the hydroxyl stretching mode gives rise to a signature at $3850\;{\text{cm}}^{-1}$, which is pronounced in the calculated Raman spectrum, but not as clear in the IR spectrum shown in figure 8*b*.

Further, on adsorption of a second hydrogen molecule, a reversible type-I hydride is formed (1.571 Å) on a single zinc ion away from the oxygen vacant site, the nature of which is confirmed by a significantly reduced energy of adsorption of 0.56 eV per molecule. Thus, terminal hydride configurations with hydrogen atoms sitting above surface zinc are identified here as the source of type-I vibrational signatures. The normal orientation of the type-I hydride to the surface is supported by an oxygen ion occupying the vacant site next to the hydride on one side and the type-II hydride, considered earlier, occupying the central vacant site on the other side. The stretching vibration of the type-I hydride is responsible for the IR and Raman signatures at $1716.97\;{\text{cm}}^{-1}$ (see figure 8*b*), which is in excellent agreement with experiment ($1710\;{\text{cm}}^{-1}$ [125,126]). In agreement with experiment, strong Raman signatures are also found for the two hydroxyl groups at 3845 and $3795\;{\text{cm}}^{-1}$ (the corresponding peak resulting from adsorption of the first hydrogen molecule undergoes only a slight red shift as a second peak arises ca. $50\;{\text{cm}}^{-1}$ below). However, only weak IR signals are calculated for these two modes—see figure 8*b*. Stabilization of the second hydride (type-I) leads to a much more significant red shift of the type-II asymmetric stretching band from $1408\;{\text{cm}}^{-1}$ to $1147\;{\text{cm}}^{-1}$ while the symmetric stretching mode has a blue shift from 960 to $986\;{\text{cm}}^{-1}$. Further, the in-plane asymmetric stretching mode of the type-II hydride has a higher IR intensity (with a 3 : 1 ratio) than that of the type-I hydride stretching normal to the surface; inversely, the type-I hydride stretching is much more pronounced in the Raman spectrum compared with the type-II hydride signal (with a 11 : 1 ratio). The symmetric type-II hydride stretch behaves similarly but with a stronger Raman signal and somewhat weaker IR signal (figure 8*b*).

Finally, dissociation of a third hydrogen molecule leads to formation of another type-I pair of a hydroxyl and a hydride, the latter with a slightly tilted bond (1.547 Å) (figure 8*c*). The stretching mode of the newly formed hydride is responsible for strong IR and Raman signals at $1801\;{\text{cm}}^{-1}$. The signature of the type-I stretching mode of the first hydride is now blue shifted to 1775 from $1717\;{\text{cm}}^{-1}$, with a relatively decreased Raman intensity. With addition of the new adsorbates, both in-plane asymmetrical and symmetrical stretching modes of the type-II Zn-H-Zn hydride experience blue shifts from 1148 to $1650\;{\text{cm}}^{-1}$ and from 986 to $1077\;{\text{cm}}^{-1}$, respectively, as the coordination bond from the bridging hydride to the third zinc ion decorating the vacant site is broken—the zinc ion moves away from the hydride in the vacancy to a separation distance of 2.774 Å, and becomes hydrogenated. Hydroxyl groups give rise to blue shifted signals at 3808, 3822 and $3840\;{\text{cm}}^{-1}$ (table 4). A similar pattern in both IR and Raman intensities is observed.

**Table 4.** Assignment of vibrational modes of hydrogen adsorbates on ZnO polar surfaces.

frequencies of adsorbed hydrogen species (${\text{cm}}^{-1}$)			
	experimental	computational	assignment
2H@ZnO			
	850 (Δ=14.5) (IR) [124]	858.80	$\delta ({\text{OH}}^{\left[1\right]})$
	1125 (INS) [125]	960.49	$\nu_{\text{s}}({\text{ZnHZn}}^{\left[1\right]})$
	1475 (Δ=300) (IR)[124]	1408.75	$\nu_{\text{as}}({\text{ZnHZn}}^{\left[1\right]})$
	3400 (Δ=300) (IR) [124]	3850.05	$\nu ({\text{OH}}^{\left[1\right]})$
4H@ZnO			
	850 (Δ=14.5) (IR) [124]	861.02	$\delta ({\text{OH}}^{\left[1\right]})$
	850 (Δ=14.5) (IR) [124]	900.13	$\delta ({\text{OH}}^{\left[2\right]})$
	1125 (INS) [125]	985.76	$\nu_{\text{s}}({\text{ZnHZn}}^{\left[1\right]})$
	1125 (INS) [125], 1475 (Δ=250) (IR) [124]	1147.94	$\nu_{\text{as}}({\text{ZnHZn}}^{\left[1\right]})$
	1708 (Δ=12) (IR) [124]	1716.97	$\nu ({\text{HZn}}^{\left[2\right]})$
	3498 (Δ=29) (IR) [124]	3795.36	$\nu ({\text{OH}}^{\left[2\right]})$
	3400 (Δ=300) (IR) [124]	3844.90	$\nu ({\text{OH}}^{\left[1\right]})$
6H@ZnO			
	850 (Δ=14.5) (IR) [124,125]	843.93	$\delta ({\text{OH}}^{\left[1\right]})$
	850 (Δ=14.5) (IR) [124]	871.63	$\delta ({\text{OH}}^{\left[2\right]})$
	850 (Δ=14.5) (IR) [124]	910.02	$\delta ({\text{OH}}^{\left[3\right]})$
	1125 (INS) [125]	1077.36	$\nu_{\text{s}}({\text{ZnHZn}}^{\left[1\right]})$
	1475 (Δ=250) (IR) [124] , 1600 (Raman) [126]	1649.91	$\nu_{\text{as}}({\text{ZnHZn}}^{\left[1\right]})$
	1708 (Δ=12) (IR) [124], 1665 (INS) [125]	1775.42	$\nu ({\text{HZn}}^{\left[2\right]})$
	—	1800.99	$\nu ({\text{HZn}}^{\left[3\right]})$
	3498 (Δ=29) (IR) [124]	3807.65	$\nu ({\text{OH}}^{\left[2\right]})$
	3498 (Δ=29) (IR) [124]	3822.46	$\nu ({\text{OH}}^{\left[3\right]})$
	3400 (Δ=300) (IR) [124]	3840.16	$\nu ({\text{OH}}^{\left[1\right]})$

Experimentally observed spectroscopic features associated with hydrogen adsorption on ZnO are collected in table 4. There is a notable agreement in assignment of type-I and type-II hydride related frequencies, including their IR intensities. Notably, the strong IR signature of type-I hydride stretching at ca. $1708\;{\text{cm}}^{-1}$ and the broad band of type-II hydride stretching motions (computed at 1147.94, 1408.75 and $1649.91\;{\text{cm}}^{-1}$) centring at ca. $1475{\text{cm}}^{-1}$, as reported from experiment (see figs. 1 and 2 and discussion section (b) in [124]), are confirmed by our calculations as shown in figure 8. The calculated bands, however, have been obtained in the harmonic approximation. The anharmonic factors would undoubtedly lead to a typical reduction in the calculated values, but for such complex species, it is difficult to predict accurate scaling factors. In the absence of zinc vacancies in this study, there are no type-II hydroxyl bands appearing in the calculated spectra. The type-I hydroxyl group signatures in this context can be assigned to all hydroxyl groups modelled [94]. Significantly, the intensity patterns are predicted in good agreement with experiment [124,125].

## 4. Conclusion

The capabilities of the hybrid QM/MM approach to reproduce computational vibrational signatures of different types of catalytic systems have been demonstrated. A strong connection, in principle, between theoretical computations and practical experiments of IR, Raman and RR spectra has been established in this work to give useful insights. The flexibility of QM/MM modelling provides valuable access to various levels of theories in multiscale modelling, with straightforward inclusion of the adiabatic environmental interactions mimicking realistic conditions from external fields. This approach therefore generates more accurately chemical predictions in large material and molecular systems, which can be calculated efficiently on high-performance computing platforms using the task-farming parallelization framework implemented in Py-ChemShell.

The new functionality was demonstrated on a diverse range of chemical systems. For solvated L-histidine, which exists in various protonation and tautomeric states, our simulated IR and Raman spectra were able to reproduce the major vibrationally active modes reported in experimental studies of histidine in aqueous solution. Simulated QM/MM RR spectra were also able to reproduce the characteristic signatures of the six-coordinated CO-bound *Ax*CYTcp haem protein, with both vibrational frequencies and relative intensities comparing well with experimental RR spectra. In application to zeolites, we have investigated adsorption of ammonia on Brønsted and Lewis acid sites, which proceed as chemisorption and physisorption, respectively, as confirmed by their spectroscopic signatures that agree with the experiment. Furthermore, a polarizable embedding model has been employed in a study of heterolytic dissociation of hydrogen over the oxygen-terminated polar surface of zinc oxide, where spectroscopic features of both reversible and irreversible modes of adsorption were unambiguously identified with the evolution of hydride surface species.

The vibrational module in Py-ChemShell continues to be actively developed. Areas of interest include isotopic effects, which can in principle be straightforwardly studied using existing data through amendment of atomic masses. Frequency calculations can then be run as a quick restart without doing any new DFT calculations, for which an automated procedure will be implemented. Bouncing effects at the boundary between the vibrational active and vibrational frozen regions in a QM/MM model also need to be addressed, as they may interfere with the accuracy of the computed vibrational spectra. The soft boundary approach is known to alleviate such problems in molecular dynamics [127,128]. Likewise as a crude device, we have in our study chosen soft species (oxygen-terminated vibrational active region) for such boundaries to limit the bouncing to a minimum level. Future work will focus on new developments of more sophisticated vibrational embedding to diminish the bouncing effects for different types of systems. Going beyond the harmonic approximation, work is also in progress on the calculation of vibrational spectra from QM/MM molecular dynamics simulations [129]. Complementary to MD-based vibrational analysis, we will develop further support for the calculation of higher-order force constants based on analytical gradients [122]. Although analytical second- and higher-order derivatives are available in computational codes, the evaluation of these quantities based on gradients allows us to study more complex models. For example, the self-consistency of shells regarding efficient polarization within or from the MM embedding environment and the point charges in the QM calculations rarely have analytical second-order derivatives. Moreover, there has been a trend in hardware with reducing memory per core for massively parallelized modern programming, whereas analytical second-order derivatives often demand much more memory and computational time.

Overall, our test cases have shown that harmonic QM/MM calculations of vibrational signatures are capable of reproducing experimental results to a good level of agreement and provide insights into the chemical processes. In some of our applications, we have observed that there are significant discrepancies between experimental and computed vibrational frequencies for some vibrational modes; a potential cause of this is neglect of anharmonic effects in our calculations. We are currently implementing more advanced techniques for calculation of vibrational spectra signatures based on vibrational self-consistent field approaches, and these effects will be evaluated in subsequent studies.

## Data Availability

The data are provided in the electronic supplementary material [130].
